# A Research Agenda for Assessment and Management of Psychosis in Emergency Department Patients

**DOI:** 10.5811/westjem.2019.1.39263

**Published:** 2019-02-19

**Authors:** Jennifer Peltzer-Jones, Kimberly Nordstrom, Glenn Currier, Jon S. Berlin, Cynthia Singh, Sandra Schneider

**Affiliations:** * Henry Ford Health System, Department of Emergency Medicine, Detroit, Michigan; † University of Colorado School of Medicine, Department of Psychiatry, Denver, Colorado; ‡ University of South Florida, Morsani College of Medicine, Department of Psychiatry, Tampa, Florida; § Medical College of Wisconsin, Department of Psychiatry and Behavioral Medicine, Department of Emergency Medicine, Milwaukee, Wisconsin; ¶ American College of Emergency Physicians, Irving, Texas; || John Peter Smith Hospital, Department of Emergency Medicine, Fort Worth, Texas; # Hofstra Northwell School of Medicine, Hempstead, New York

## Abstract

**Introduction:**

Emergency departments (ED) manage a wide variety of critical medical presentations. Traumatic, neurologic, and cardiac crises are among the most prevalent types of emergencies treated in an ED setting. The high volume of presentations has led to collaborative partnerships in research and process development between experts in emergency medicine (EM) and other disciplines. While psychosis is a medical emergency frequently treated in the ED, there remains a paucity of evidence-based literature highlighting best practices for management of psychotic presentations in the ED. In the absence of collaborative research, development of best practice guidelines cannot begin. A working group convened to develop a set of high-priority research questions to address the knowledge gaps in the care of psychotic patients in the ED. This article is the product of a subgroup considering “Special Populations: Psychotic Spectrum Disorders,” from the 2016 Coalition on Psychiatric Emergencies first Research Consensus Conference on Acute Mental Illness.

**Methods:**

Participants were identified with expertise in psychosis from EM, emergency psychiatry, emergency psychology, clinical research, governmental agencies, and patient advocacy groups. Background literature reviews were performed prior to the in-person meeting. A nominal group technique was employed to develop group consensus on the highest priority research gaps. Following the nominal group technique, input was solicited from all participants during the meeting, questions were iteratively focused and revised, voted on, and then ranked by importance.

**Results:**

The group developed 28 separate questions. After clarification and voting, the group identified six high-priority research areas. These questions signify the perceived gaps in psychosis research in emergency settings. Questions were further grouped into two topic areas: screening and identification; and intervention and management strategies.

**Conclusion:**

While psychosis has become a more common presentation in the ED, standardized screening, intervention, and outcome measurement for psychosis has not moved beyond attention to agitation management. As improved outpatient-intervention protocols are developed for treatment of psychosis, it is imperative that parallel protocols are developed for delivery in the ED setting.

## INTRODUCTION

Psychosis is an important clinical problem, not only for patients but for families and healthcare workers as well. Patients with mental disorders represent an increasing fraction of total presentations to emergency departments (ED) over time. In 2014, mental disorders were the 10^th^ leading cause of United States ED visits for males aged 15–65 years, and mental disorders were the primary ED diagnosis in slightly over five million ED visits.^1^ Thus, development of better management approaches to assess and treat psychosis has become critical. With other high volume/high risk medical emergencies – traumatic injuries, cerebrovascular accidents, cardiac arrhythmias – emergency medicine (EM) has been able to partner with other medical specialties to jointly research and develop best practice care. However, translation of best practice care of psychosis specific to an emergency setting has yet to occur.

Mounting evidence suggests early intervention predicts improved outcomes in younger, first-episode psychotic patients. Yet to our knowledge, no evidence-based interventions linking first-episode psychotic ED patients into specialized treatment have been tested. This deficit highlights the collaborative treatment chasm between mental health and ED specialty fields. Appropriate recognition, categorization and management of psychosis should be a key element of comprehensive emergency care; achieving these goals can be done through improved mental health and emergency care collaboration. The goal of this research consensus workgroup was to explore and enumerate the current knowledge gaps for the care of psychosis specifically in an emergency setting.

## METHODS

Participants from a variety of disciplines – EM, emergency psychiatry, emergency psychology, clinical research, governmental agencies, and patient advocacy groups – were invited to participate in a research consensus session held prior to a joint emergency-psychiatry conference (the 7^th^ Annual National Update on Behavioral Emergencies). Background literature reviews were performed prior to the in-person meeting. Literature reviews were conducted via journal review, academic databases and web-based searches. Searches fell within the scope of the priority domain identified by the Coalition on Psychiatric Emergencies (CPE) steering committee: acute psychosis. The workgroup leaders identified articles of importance and circulated them electronically to the group for review in advance of the in-person meeting. A nominal group technique^2^ was employed to develop group consensus on the highest priority research gaps. Following the nominal group technique, input was solicited from all participants during the meeting, questions were iteratively focused and revised, voted on, and then ranked by importance. Following the in-person meeting, the workgroup developed additional consensus and worked electronically to further refine the final form of each question. Please see the Executive Summary for the full methods (Appendix).

## RESULTS

The group consisted of three emergency psychiatrists, an emergency psychologist, an emergency physician, clinical researcher and participant from a professional medical association. The average age of the participants was approximately 40 years old and included five females and two males. The group developed 28 separate questions. After clarification and voting, the group identified six high-priority research areas. Questions were further grouped into two topic areas: screening and identification; and intervention and management strategies. The questions organized by topic, are included in Table 1 and 2.

## DISCUSSION

This discussion highlights current knowledge gaps and rationale as to why improved patient care processes cannot be implemented until this research is conducted.

### Question 1: Can a research-based triage tool be developed to assess psychosis in ED patients?

Psychosis is a symptom rather than a definitive diagnosis, and it is a continuous rather than a categorical phenomenon. At one extreme, patients can be quietly delusional and at risk of self-harm and at the other extreme, paranoid with poor reality testing posing an extreme and immediate risk to ED staff and other patients. While rating scales for psychosis such as the Brief Psychiatric Rating Scale have been employed for over 50 years and assess several domains of psychosis, they have not been incorporated into ED care.^3^ The ED-based scales that have been developed and normed tend to focus primarily on agitation as the primary outcome and not on the variety of psychosis symptoms.

Tools assessing positive and negative domains of psychosis have not been standardized as valid or reliable within an ED setting.^4^ This deficit has led to a misunderstanding of the true incidence and prevalence of psychosis presentations in EDs. Without better, clearer definitions, ED and mental health providers will continue to have a chasm in care. Articulation of a more refined definition of psychosis that is measurable, relevant to ED care, understandable to ED providers, and captures the most salient symptoms of psychosis should be a high priority on any research agenda to ensure that both mental health and emergency providers are sharing a common language. Creation of such tools can then guide goal-directed treatment strategies within the ED.

An additional difficulty with psychosis presentations to an ED is the heterogeneous etiologies that can produce episodes of psychoses. Psychotic presentations are not all related to underlying mental illness (e.g., postictal states, metabolic derangements, substance intoxication/withdrawal, etc.). Because of this, there is a need to employ organized evaluations of psychosis. Using standardized algorithms would improve correct etiology identification and lead to proper treatment choices. For example, up to half of patients presenting to psychiatric EDs have concurrent substance use disorder. Schanzer et al. found ED clinicians inaccurately ascribed first presentations of psychosis to primary psychiatric disorders instead of substance misuse in one quarter of patients evaluated.^5^ This type of inaccurate diagnosing creates missed opportunities for chemical dependency interventions and leads to referral of patients to the wrong levels of care. Standardized ED medical evaluation algorithms for psychosis have been published in academic literature and adopted in several states, which help ED staff detect primary psychotic disorders from medical mimics.^6^–^11^ Without universal adaptation of medical evaluation protocols for psychotic presentations, there is a continued risk of misidentifying mental health etiology from medical etiology, leading to inappropriate or missed interventions.

### Question 2: What outcomes are meaningful for patients/families when assessing the effectiveness of psychosis interventions?

At present, literature regarding emergent psychosis intervention has predominantly focused on management of agitation.^12^–^15^ First- or second-generation antipsychotic medication interventions have measured outcomes such as achieving calm behavior^16^ or decreasing need for additional medications.^15^,^17^ These measures neglect the vast spectrum of distressing, patient-level experiences of psychosis such as delusional thought content, sensory hallucinations, and negative affective states. While agitation can be a symptom of psychosis, agitation is not a pathognomonic symptom for psychosis; thus, efficacy of psychosis interventions must be broadened. While it is possible there is a direct link between treatment of agitation and alleviation of patient symptoms, further research in this field is needed. Because the bulk of literature has focused on management of agitation, it is not well known what the most important outcomes are for psychosis intervention in the ED according to patients and families. The effects of emergency intervention care choices relative to patient/family satisfaction, patient quality of life, patient course of illness, future patient/family crisis help seeking, etc., is also largely unknown. Additional patient- and family-centered studies in this area are necessary.

### Question 3: What is the recommended treatment for psychosis in the emergency setting?

There is mounting evidence that early and aggressive intervention for first-episode psychosis (FEP) related to schizophrenia makes a significant impact on longer term outcomes.^18^ Since many patients with FEP present initially to the ED rather than to mental health treatment settings, opportunities to link patients into care are dependent upon the knowledge base of the ED providers. As compared to other medical disorders treated in the emergency setting, there is a significant deficit in best practice interventions for first, or subsequent, episodes of psychosis. At least one randomized, controlled trial demonstrated the superiority of outpatient, multimodal treatment strategies for FEP as compared to treatment as usual,^19^ but how similar interventions can be developed for an emergency setting is unclear. More specifically, while recommendations for psychosis treatment are available in psychiatric literature, no studies have yet standardized the education and engagement of these non-ED best practice recommendations such as medication management, family psychoeducation, social skills training, and supported employment/education programs, into emergency care protocols.^19^,^20^ Therefore, it is not known if rapid linkage to specialized outpatient treatment can improve outcomes.

It could be argued that the lack of standardized algorithms for new onset psychosis care as compared to interventions for other newly diagnosed disease states, such as diabetes, represents both a healthcare disparity in how mental illness is managed and a chasm in collaborative care between emergency and mental health researchers. As programs for earlier identification and intervention (i.e., prodromal presentations) are implemented nationally and internationally, it is not well defined as to how emergency providers will receive education and training to identify individuals at risk and provide recommended care.^21^ In addition to management of FEP, it is unclear what best practice emergency guidelines are for psychosis decompensation along the life course of the illness. It is not known if psychotic presentations in the first three years of an illness should be targeted and treated differently than in later years.

Aside from medication strategies, little research has been conducted investigating non-pharmacologic interventions for psychosis in the ED. Psychotic patients in the ED have a wide variety of behavioral presentations, often with subtle but important variations. For example, agitated patients may self-present seeking appropriate and effective medication for their condition, or they may be brought in involuntarily because of resistance to treatment, hostility, paranoia, and physical aggression. Often the literature on psychotic agitation does not distinguish between these two presentations and focuses on selecting an appropriate medication and route of medication for agitation. However, the importance of engagement, collaboration and, specifically, the art of engaging the individual around medication is key.^22^

Psychiatric emergency service (PES) practitioners note a significant reduction in outcomes such as decreased use of restraint and seclusion, as well as increased safety to both staff and patient, when the attempt to form a therapeutic alliance is prioritized.^23^ PES refers to specialized psychiatric crisis response centers and are not housed within EDs, generally managed by psychiatrically trained staff. ED providers may not receive the same training on building therapeutic alliances with patients as compared to mental health practitioners. It is unclear if providing increased education to ED providers on enhancing patient alliance could lead to improved ED patient engagement, as these types of outcome studies have not been conducted.

### Question 4: What affects emergency provider decision-making in treatment choice for psychosis?

In a recent longitudinal review, Bessaha’s group highlighted the lack of standardized clinical protocols when they examined disposition decisions for psychotic illness presentations.^24^ There were significant differences in hospitalization rates dependent upon non-clinical factors such as race, gender, and geographic location, although why these differences exist is unknown. How patients present to emergency settings, what resources are available to them, the level of emergency provider training in behavioral health assessment, and familiarity with psychopharmacology principles all may ultimately contribute to the disposition decision-making of the emergency provider. It is not understood how the interplay between patient severity level and non-patient factors combines to determine treatment decisions. It is unclear if these decisions are efficacious in illness management.

### Question 5: What system outcomes can be affected by early treatment of psychosis in emergency settings – both within emergency care settings and thereafter?

While earlier questions focused on patient-centered outcomes, it is not known if evidenced-based care can positively affect system-level outcomes such as ED throughput. Nationally, there is recognition that patients with mental health complaints have longer ED lengths of stay (LOS) than those presenting without mental health complaints.^25^ More specifically, patients who present in mental health crisis and who have a diagnosis of psychosis have longer ED LOS than patients without mental health complaints.^16^,^26^ At present, knowledge gaps exist in how often a patient receives an intradepartmental intervention, how early into an emergency presentation patients receive treatment, and whether earlier intradepartmental interventions can make a difference in disposition choices. These metrics are not monitored in the same way EDs deliver interventions such as early goal-directed treatment of sepsis, time to cardiac catheterization, or door to needle time for cerebrovascular accidents. Creating evidenced-based guidelines and metrics for acute mental illness should mimic acute medical disorder protocols. Because we do not have a standard, goal-directed psychosis treatment algorithm, it is unclear if early treatment can affect ED throughput, subsequent inpatient psychiatric LOS, or safety outcomes (i.e., use of restraints/seclusion or patient/family/provider injury).

### Question 6: Are there appropriate care locations for psychotic patient presentations instead of the ED?

With increasing alternative models of care – specifically PES – it is not fully known how these settings can contribute to better patient or system outcomes. Mental health systems of care do not have standardized formulas on which to base decisions about developing new facilities, and PES are not all developed and accessed in the same way. How PES care enhances psychosis management differently than general ED care as it relates to patient- and system-level outcomes is unknown. For example, in comparing PES services with general EDs, which site provides more consistent psychosis interventions, which site is better able to serve first-onset psychosis vs safety net concerns such as medication refills; which site works better with non-mental health professionals (such as emergency medical services, or police)? Additional research is needed to compare and contrast psychosis outcomes between these differing models of care.

## LIMITATIONS

There are several limitations to this study. First, this was not an empirical literature review, but rather an expert group of research clinicians and others who engaged in a nominal group technique to come to a consensus on setting future research priorities for the management of psychosis in the ED based on the knowledge of the current gaps in existing literature. Due to the lack of existing literature on psychosis management in the ED, the two articles sent for review prior to the conference focused on early interventions for psychosis in the community setting.^19^, ^21^ By the time this paper is published, it is possible studies may have been conducted that focus on the gaps in knowledge outlined through this research consensus conference. An additional limitation includes use of the nominal group technique, as it is different from large literature reviews/meta-analytic studies, which highlight what is known. This meeting and subsequent discussion focused on gaps in literature in order to set a future research agenda focused on psychosis management in the ED. Discussing what does not exist vs what is known could be perceived as a limitation. Our hope is that in highlighting what is missing from current literature, we can help shape research agendas moving forward.

Another limitation was in the psychosis workgroup selection. While the group engaged a variety of practitioners from emergency settings, it was limited to emergency specialists. One could argue that the inclusion of important stakeholders, such as inpatient psychiatric clinicians, could have provided additional perspectives on what areas are of highest priority to explore. Lastly, the group focus was narrowed to primary psychosis and did not include psychotic presentations due to substance intoxication/withdrawal or medical etiologies. We excluded substance-related psychosis presentations because we knew that a different group at this conference, which focused on substance-related presentations, was performing an identical critical review. Identification of psychotic presentations due to underlying medical problems has been extensively discussed in the literature in the context of the ongoing medical clearance work. The group felt it was of greater impact to focus on primary psychotic illness management, which has not had the same type of attention and focus in the research literature.

## CONCLUSION

EDs are increasingly expected to provide interventions for acute psychosis, both for first episodes of psychosis or during exacerbations of chronic illness, yet there are no current, evidence-based protocols for treatment of psychosis care. Addressing the identified research questions would serve as first steps in developing standardized algorithms for psychosis care and improving treatment in the ED setting.

## Supplementary Material



## Figures and Tables

**Table 1:** Key research questions to guide efforts for individuals with psychosis through screening and identification.

Question 1	Can a research-based triage tool be developed to assess psychosis in ED patients?
Question 2	What outcomes are meaningful for patients/families when assessing the effectiveness of psychosis interventions?

*ED*, emergency department.

**Table 2:** Key research questions to guide efforts for effective intervention and management of the patient with acute psychosis.

Question 3	What is the recommended treatment for psychosis in the emergency setting?
Question 4	What affects emergency provider decision-making in treatment choice for psychosis?
Question 5	What system outcomes can be affected by early treatment of psychosis in emergency settings - both within the emergency care setting and thereafter?
Question 6	Are there appropriate care locations for psychotic patient presentations instead of the ED?

*ED,* emergency department.
